# Neuroanatomical correlates of genetic risk for obesity in children

**DOI:** 10.1038/s41398-022-02301-5

**Published:** 2023-01-03

**Authors:** Filip Morys, Eric Yu, Mari Shishikura, Casey Paquola, Uku Vainik, Gideon Nave, Philipp Koellinger, Ziv Gan-Or, Alain Dagher

**Affiliations:** 1 Montreal Neurological Institute, McGill University, Montréal, Canada; 2 Department of Human Genetics, McGill University, Montréal, Canada; 3 Institute of Neuroscience and Medicine (INM-1), Forschungszentrum Jülich, Jülich, Germany; 4 Institute of Psychology, Faculty of Social Sciences, University of Tartu, Tartu, Estonia; 5 Institute of Genomics, Faculty of Science and Technology, University of Tartu, Tartu, Estonia; 6 Marketing Department, The Wharton School, University of Pennsylvania, Philadelphia, USA; 7 Department of Economics, School of Business and Economics, Vrije Universiteit Amsterdam, Amsterdam, The Netherlands; 8 Department of Neurology and Neurosurgery, McGill University, Montréal, Canada

**Keywords:** Neuroscience, Diseases, Genetics

## Abstract

Obesity has a strong genetic component, with up to 20% of variance in body mass index (BMI) being accounted for by common polygenic variation. Most genetic polymorphisms associated with BMI are related to genes expressed in the central nervous system. At the same time, higher BMI is associated with neurocognitive changes. However, the direct link between genetics of obesity and neurobehavioral mechanisms related to weight gain is missing. Here, we use a large sample of participants (*n* > 4000) from the Adolescent Brain Cognitive Development cohort to investigate how genetic risk for obesity, expressed as polygenic risk score for BMI (BMI-PRS), is related to brain and behavioral measures in adolescents. In a series of analyses, we show that BMI-PRS is related to lower cortical volume and thickness in the frontal and temporal areas, relative to age-expected values. Relatedly, using structural equation modeling, we find that lower overall cortical volume is associated with higher impulsivity, which in turn is related to an increase in BMI 1 year later. In sum, our study shows that obesity might partially stem from genetic risk as expressed in brain changes in the frontal and temporal brain areas, and changes in impulsivity.

## Introduction

Obesity is consistently associated with alterations in brain anatomy [1–10]. These changes can be detected by magnetic resonance imaging (MRI) and affect both grey and white matter. Numerous mechanisms have been proposed to explain these findings.

Brain morphometry may underpin behavioral traits that predispose to obesity [11–14]. Indeed, obesity is heritable and polygenic [15–18] - twin studies show that the heritability of body mass index (BMI) is 70%, and large-scale meta-analyses of genome-wide association studies (GWAS) have identified over 700 risk alleles [15, 18–20]. Polygenic risk scores for BMI (BMI-PRS), representing the sum of genetic risk per individual, account for 5–15% of variance in BMI proper [18, 21]. It has been suggested that the risk alleles act in the brain to cause a behavioral phenotype that renders individuals prone to positive calorie balance and weight gain [17, 22, 23]. This phenotype may involve satiety and hunger signaling in the hypothalamus, but may also include the trait uncontrolled eating, which has been linked to function of brain systems implicated in learning and memory, stress, motivation, and executive control [14, 15, 23, 24].

Conversely, it is also known that obesity can change the brain. Chronically, adiposity is associated with a metabolic syndrome that includes hypertension, hyperlipidemia, and insulin resistance, which can cause brain atrophy over time [25]. Even over shorter time-spans, positive calorie balance may lead to adaptive brain changes. Studies of diet-induced obesity in rodents show widespread alterations in synaptic density and neuronal composition, occurring in parallel with or even prior to weight gain [26–33].

Disentangling the chain of causality between brain anatomy and obesity is difficult. The approach used here is to estimate the effect of polygenic risk for obesity on brain anatomy in young children. This would be expected to limit the effect of potential confounds due to metabolic syndrome and have a better chance to identify the inherited neural endophenotype that renders individuals vulnerable to weight gain. We focus on genetic potential for adult obesity, as the goal of childhood obesity prevention should be avoidance of adult obesity, which is causally linked with negative health outcomes [34]. Here, we analyze how genetic risk for adult obesity is associated with brain structure, executive function, impulsivity, and 1-year BMI change in a sample of 4157 children aged 9–11 years.

## Materials and methods

### Participants – main sample (ABCD)

We used data from the Adolescent Brain Cognitive Development Cohort (ABCD), a longitudinal, multi-site study from the USA [35–37]. Study procedures were approved by review boards of all participating sites and written parental informed consent and child assent were collected prior to participation. We excluded participants with outlier BMI values (below 10 kg/m^2^ or above 50 kg/m^2^) [11]. We only included participants with full neuroimaging data that passed all quality control checks. The final sample consisted of 4,157 children of European descent (mean age = 10 years, SD = 0.5 year; mean BMI = 17.94 kg/m^2^, SD = 3.30 kg/m^2^; mean weight = 35.60 kg, SD = 8.58 kg; 1940 girls). At the present time PRS calculation is only possible in individuals of European descent as GWAS were conducted in this population. BMI values were converted to standard deviation scores (BMI SDS), which are z-scores derived from age and sex of each participant, based on Centers for Disease Control and Prevention growth charts [38] using the ‘childsds’ package in R (mean BMI SDS = 0.20 kg/m^2^, SD = 1.09 kg/m^2^). 1-year BMI SDS change values were calculated as the difference between BMI SDS at a follow-up appointment and BMI SDS at the time of brain and behavioral data collection, hence higher values represent a BMI SDS increase.

### Neuroimaging data – main sample (ABCD)

Data were collected using 3 T magnetic resonance imaging (MRI) scanners of different manufacturers (Siemens, General Electric, and Philips) at 22 different sites. Data collection was harmonized across all acquisition sites by using standardized hardware (e.g., head coils) and adjusting acquisition sequences for each scanner manufacturer. All imaging protocols can be found elsewhere [35]. We used cortical thickness, cortical volume, and fractional anisotropy data provided by the ABCD initiative [39]. These brain phenotypes are most commonly used in studies investigating the associations between obesity and brain structure [40]. In addition, FA is considered a summary measure of white matter microstructural integrity [40, 41]. Cortical thickness and volume data for each parcel of the Desikan-Killiany (DK; 68 parcels) atlas [42] were obtained using FreeSurfer 5.3.0 [43] after correcting for gradient nonlinearity distortions. Custom scripts were used to obtain fractional anisotropy data for 35 major white matter tracts segmented using AtlasTrack [44]. Visual inspection of processed data was conducted to ensure that only images with no processing errors were included in the dataset. We used quality check values provided by the ABCD Study (pass/fail) to only include participants who passed quality control in our final sample. Prior to all but brain age analyses, we used ComBat harmonization software to remove site variability from cortical thickness, volume, and FA data [45]. In addition, we scaled all volumetric measures by total intracranial volume.

### Polygenic risk score calculation

We assessed genetic risk for obesity using BMI-PRS, which allows us to calculate each individual’s PRS from a well-powered, recent BMI GWAS [18]. BMI-PRS is reflective of a more general risk for obesity and cardiometabolic comorbidities and correlates well with body fat percentage [18, 46, 47]. BMI-PRS was calculated using PRSice-2 [48] with pruning and thresholding of BMI GWAS summary statistics from an independent data sample (https://www.ebi.ac.uk/gwas/downloads/summary-statistics) [18]. First, variants in the GWAS summary statistics were pruned based on linkage disequilibrium of variants within 250 kb and r^2^ > 0.1. Then, the p-value threshold where the PRS was most correlated with phenotypical BMI in the investigated dataset (ABCD) was chosen for PRS calculation. We normalized all BMI-PRS for downstream analyses. GWAS sample and ABCD sample are independent.

### Participants – brain age sample (PING)

To place the anatomical findings in the context of expected neurodevelopmental stage, we used a separate sample of similar age. We created a predicted brain age model to which we compared each ABCD participant’s grey matter measures. To this end, we used the Pediatric Imaging, Neurocognition, and Genetics (PING) data [49]. This dataset encompasses 1493 children aged 3–20 years collected across multiple sites in the US. Written informed parental consent and child assent was obtained for all participants aged below 18, and written consent was obtained for all participants above 18. The study procedures were approved by the review boards of all participating sites. For our analysis, we selected a subsample of participants of European descent with brain measures and age data available (*n* = 423; 196 girls). The average age of this sample was 12 years (SD = 5 years), and range was 3 to 21 years.

### Neuroimaging data – brain age sample (PING)

Data were collected using 3 T MRI scanners from GE, Siemens, or Philips. Data collection was harmonized across 10 study sites by adjusting acquisition sequences. Details of data acquisition and processing can be found in [49]. Here, we used grey matter volume and thickness measures provided by the PING consortium, as derived from FreeSurfer [43].

### Executive function and impulsivity measures

To relate our findings to behavioral measures, we used executive function and impulsivity indices, as both were previously related to obesity [50, 51]. Impulsivity was assessed using the child version of the Urgency, Premeditation, Perseverance, Sensation Seeking, Positive Urgency scale (UPPS-P [52, 53]). Here, we selected positive and negative urgency measures as they were previously associated with eating behavior [54, 55]. To assess executive function, similarly to [56], we calculated a composite score based on the results of five tests, namely the Flanker inhibitory control and attention test, the dimensional change card sort test, the picture sequence memory test, the list sorting working memory test, and the pattern comparison processing speed test [56–60] (correlations with the composite score: Flanker test: 0.64; card sort test: 0.71; picture memory test: 0.58; working memory test: 0.59; processing speed test: 0.72; all *p*-values < 0.001). Age-corrected scores were used and a composite score for each participant was derived by averaging the five standardized scores.

### Data analysis

Statistical analyses were conducted using R (v. 3.6.1; [61]). Scripts used to analyze data are available at https://github.com/FilipMorys/OBPRS.

### Relationship between BMI-PRS and phenotypical BMI SDS

We first conducted a proof-of-concept analysis to investigate whether BMI-PRS was related to measured BMI SDS. To this end, we used regression analysis with BMI SDS as an outcome variable and BMI-PRS, the first 20 genetic principal components (to control for population stratification), age, sex, interaction of age and sex, and study site as predictor.

### Relationship between brain volume and BMI-PRS

Next, we explored the relationship between genetic risk for obesity (BMI-PRS) and brain measures of interest—cortical thickness, cortical volume, and fractional anisotropy. We ran separate permutation-based regression analyses (10,000 permutations, ‘lmPerm’ package in R) for each cortical parcel and white matter tract. We used BMI-PRS as a predictor of brain measures, while accounting for age, sex, parental education, parental income, parental marital status, child’s education, and first 20 genetic principal components. We used Benjamini-Hochberg correction to adjust for multiple comparisons [62]. This was applied separately to cortical thickness, cortical volume, and white matter measures. Brain plots were prepared using ‘fsbrain’ package in R [63].

### Cortical architectonic types

To gain a better understanding of the cortical areas affected by BMI-PRS, we investigated how they relate to brain cortical profiles of the organization of cortical types along sensory processing hierarchies (i.e. idiotypic, unimodal, heteromodal, or paralimbic cortex). We used cortical architectonic maps representing the sensory-fugal gradient [64–66] and parcellated them using the DK atlas [42]. Next, we calculated total cortical grey matter volume and average cortical thickness of each of the cortical types for each subject and regressed these measures against BMI-PRS using previously described covariates—sex, age, parental education, parental income, parental marital status, child’s education, and first 20 genetic principal components (permutation-based regression, 10,000 permutations). We then calculated effect sizes (partial eta squared) to compare which architectonic types were most strongly associated with BMI-PRS cortical patterns.

### Brain age analysis

Brain maturation in adolescence is associated with changes in grey and white matter. We therefore related the neuroanatomical effects of BMI-PRS to brain age in the ABCD sample based on a model from a different dataset. This model was based on the PING sample and used grey matter measures while correcting for intracranial volume. We then used the model to estimate the brain age of participants in the ABCD sample. The model was derived using linear regression with 10-fold cross validation. Due to low sample size in the PING dataset, the model was based on global measures of brain structure: cortical grey matter volume, and average cortical thickness, rather than individual DK parcels. Prior to the analysis, the effects of sex, study site, parental income, parental education, child’s education, and parental marital status (only in ABCD) were removed from the brain measures from both ABCD and PING using linear regression. Model performance was assessed using root mean square error (RMSE). We initially included linear and quadratic terms in the model, however, since the model did not improve significantly with quadratic terms, we decided to only use linear terms in the final estimation.

Brain age for each participant in the ABCD dataset was estimated based on imaging features and the model described above. For each participant, we calculated a difference between chronological age and brain age—delta age [67, 68]. Finally, we ran a permutation-based linear regression between delta age and BMI-PRS (corrected for first 20 genetic principal components; 10,000 permutations). Because delta age is correlated with chronological age, we added chronological age to the regression as a covariate of no interest [67].

### Structural equation model

To pool our results in one model and investigate how BMI-PRS can affect BMI SDS change via brain and behavioral changes, we used a structural equation model (SEM) with the following measures of interest: BMI-PRS, global brain measures—cortical thickness, cortical volume, and FA—executive function and impulsivity, and 1-year change in BMI SDS. Data were first residualized to remove variance related to age, sex, parental education, parental marital status, household income, child’s education, study site, and first 20 genetic principal components. Additionally, BMI SDS change was residualized for baseline BMI SDS to make sure that the effects found in the SEM were not due to correlations of our variables of interest with baseline BMI SDS.

Using the lavaan package in R (version 0.6–5 [69]) we created an SEM, where we hypothesized that BMI-PRS would affect average cortical thickness, total grey matter volume, and average FA. These measures were then hypothesized to affect impulsivity and executive function, which would in turn affect BMI SDS. We allowed for residual correlations between impulsivity and executive function measures, and between brain measures.

We estimated the model using maximum likelihood estimation with pairwise missing values exclusions and robust standard errors. Model fit was assessed using root mean square error of approximation (RMSEA), standardized root mean square residual (SRMR), and comparative fit index (CFI).

### Follow-up analysis on impulsivity and regional brain volumes

Due to the presence of a significant relationship between impulsivity and global brain volume (which was also related to BMI-PRS), we followed-up on these associations and investigated the relationship between impulsivity and regional brain volume. To this end we ran separate permutation-based regression analyses (10,000 permutations) for each cortical parcel. We used positive/negative urgency as predictors of brain measures, while accounting for age, sex, parental education, parental income, parental marital status, child’s education, and first 20 genetic principal components. We used Benjamini-Hochberg correction to adjust for multiple comparisons [62]. This was applied separately to regressions investigating positive and negative urgency.

## Results

### Relationship between BMI-PRS and phenotypical BMI SDS

**Fig. 1:**
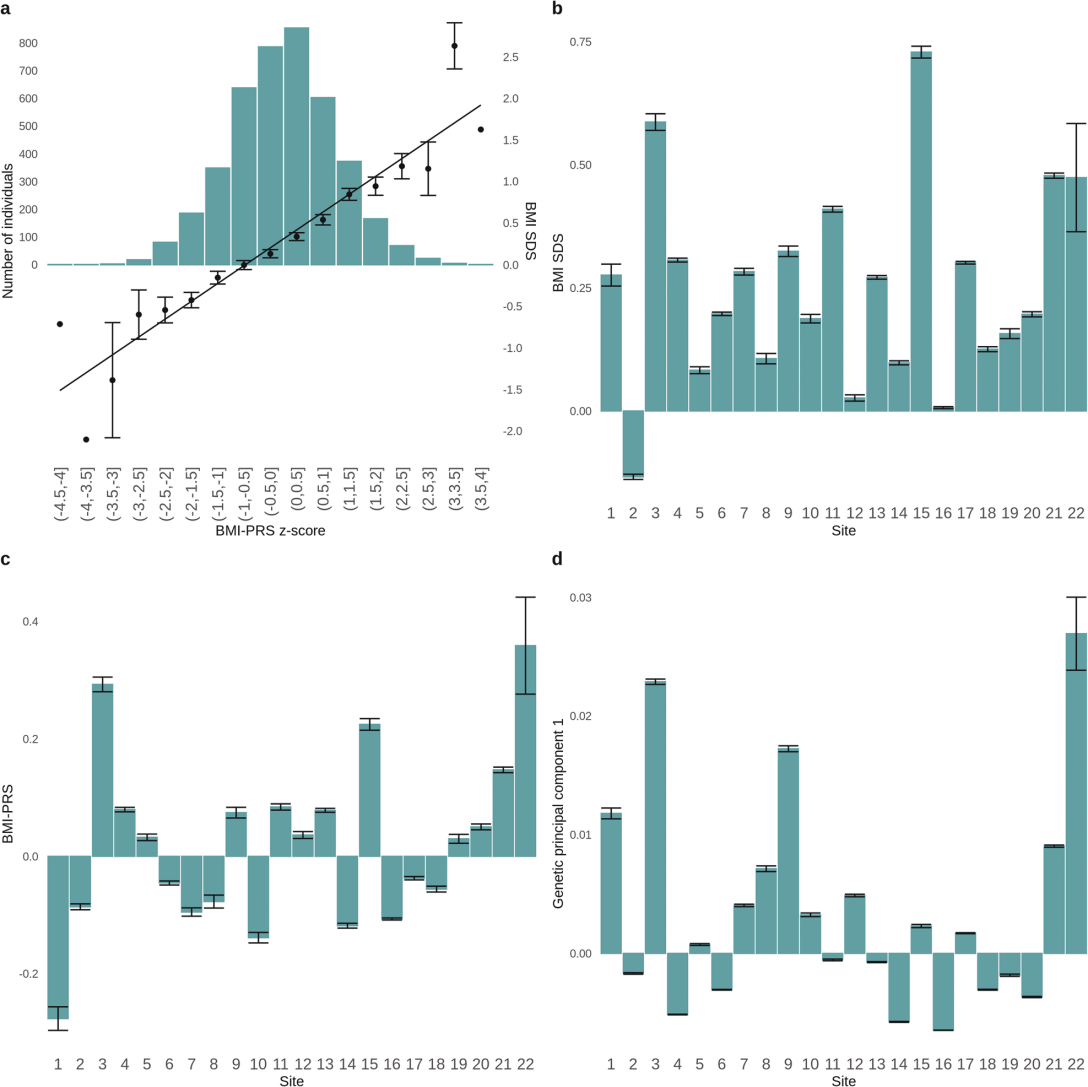
Sample and site characteristics. **a** Relationship between BMI-PRS z-score and BMI SDS (right axis) overlayed on a histogram (left axis). The histogram represents the number of participants in each BMI-PRS z-score bin; **b** relationship between BMI SDS and study site; **c** relationship between BMI-PRS and study site; **d** relationship between the first genetic principal component and study site. BMI-PRS: body mass index polygenic risk score. BMI SDS: body mass index standard deviation score.

BMI-PRS was significantly positively associated with BMI SDS (standardized beta coefficient estimate = 0.263, t = 17.610, F(45,4111) = 10.39, 95% confidence intervals: 0.255–0.319, *p* < 0.001; Fig. 1a). BMI-PRS accounted for 7.2% variance in BMI SDS beyond the first 20 genetic principal components, age, sex, study site, and age × sex interaction. As for the adults in Locke et al. [15], the BMI-PRS SNPs were normally distributed in our population, and the effect of risk SNPs on BMI was additive (Fig. 1a). BMI SDS, BMI-PRS, and genetic principal components strongly differed by study site (Fig. 1b–d), hence all the results presented here are corrected for study site and genetic principal components unless otherwise stated.

### Brain-PRS relationship

**Fig. 2:**
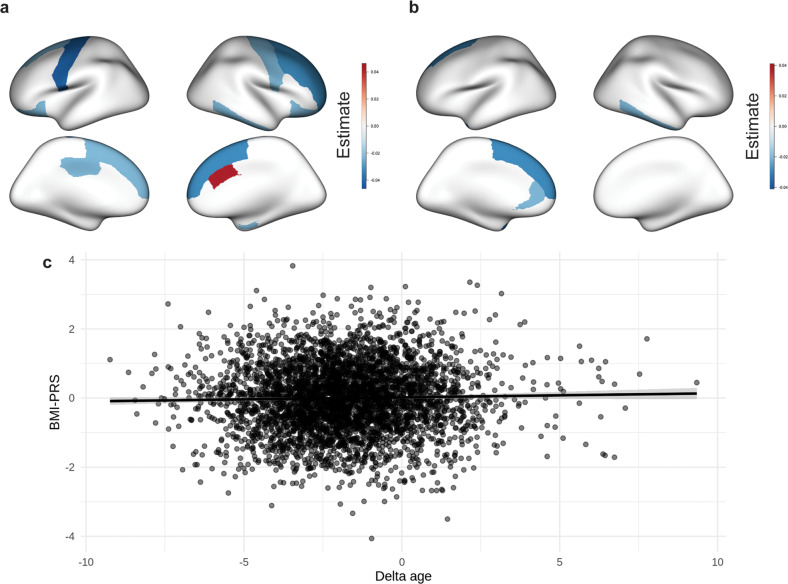
Genetic risk for obesity and brain outcomes. **a** Relationship between BMI-PRS and cortical volume; **b** relationship between BMI-PRS and cortical thickness; **c** relationship between BMI-PRS and predicted brain age.

BMI-PRS was negatively associated with cortical volume in several brain areas, predominantly in the frontal and temporal lobes (Table 1, Fig. 2a). The strongest associations were visible in the left precentral and right superior frontal gyri. BMI-PRS was also associated with lower cortical thickness in the frontal and temporal areas (Table 1, Fig. 2b). We did not find any significant associations between BMI-PRS and white matter fractional anisotropy.

**Table 1:** Relationship between BMI-PRS, cortical thickness, and cortical volume.

Parcel	Hemisphere	Regression estimate	*p*-value
Cortical volume			
Lateral orbitofrontal	Left	−0.024	<0.001
Posterior cingulate		−0.023	<0.001
Precentral		−0.046	<0.001
Superior frontal		−0.020	<0.001
Caudal anterior cingulate	Right	0.040	<0.001
Caudal middle frontal		−0.035	<0.001
Entorhinal		−0.023	<0.001
Inferior temporal		−0.028	<0.001
Lateral orbitofrontal		−0.025	<0.001
Precentral		−0.029	0.012
Rostral middle frontal		−0.031	<0.001
Superior frontal		−0.037	<0.001
Cortical thickness			
Rostral anterior cingulate	Left	−0.025	<0.001
Superior frontal		−0.033	<0.001
Temporal pole		−0.041	<0.001
Inferior temporal	Right	−0.023	<0.001

### Cortical architectonic types

To investigate whether cortical volume and thickness changes related to BMI-PRS belong to specific cortical architectonic types defined by Mesulam [64], we calculated total cortical volume and average cortical thickness for each of the types (idiotypic, unimodal, heteromodal, and paralimbic) per subject and investigated their association with BMI-PRS. Total cortical volume of the heteromodal and unimodal cortex, but also average cortical thickness of the heteromodal cortex were significantly negatively associated with BMI-PRS (Table 2).

**Table 2:** Association between cortical thickness/volume for each cortical architectonic type and BMI-PRS.

Cortical type	Partial η^2^	Regression estimate	*p*-value
Cortical volume			
Idiotypic	0.0001	−0.103	1.000
Unimodal	0.0008	−0.203	<0.001
Heteromodal	0.0012	−0.252	<0.001
Paralimbic	0.0002	−0.104	0.106
Cortical thickness			
Idiotypic	0.0002	0.009	0.959
Unimodal	0.0001	−0.008	0.574
Heteromodal	0.0004	−0.012	0.033
Paralimbic	0.0002	−0.009	1.000

### Brain age analysis

The grey matter-based brain age model returned an RMSE of 3.56 years. Higher brain age was related to lower cortical thickness and volume. Regression analysis showed that grey matter-related delta age in the ABCD dataset was significantly positively related to BMI-PRS (estimate = 0.009, *p* = 0.034; Fig. 2c). This indicates that higher genetic risk for obesity is associated with cortical thinning and volume decreases, relative to age-expected values.

### Structural equation model

**Fig. 3:**
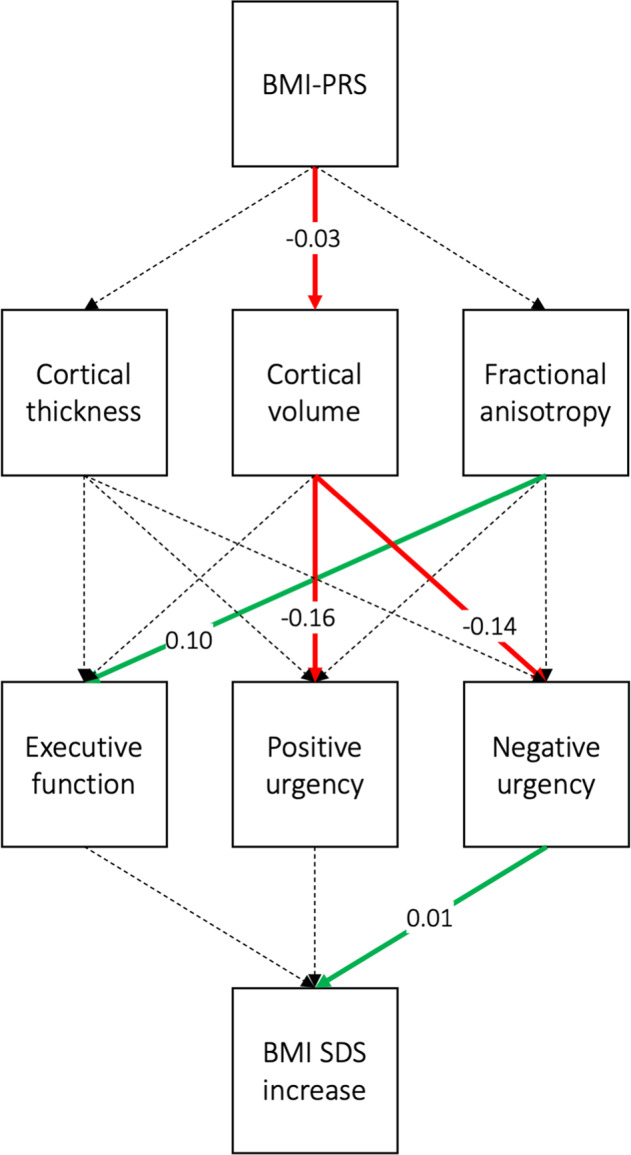
Schematic representation of the structural equation model. Significant associations are marked in red/green, non-significant associations are marked with dashed lines. BMI-PRS body mass index polygenic risk score. BMI SDS body mass index standard deviation score.

The structural equation model returned a good fit (RMSEA = 0.041, CFI = 0.979, SRMR = 0.020; *χ*^2^ = 55.349, *p* < 0.001). We found significant negative associations between BMI-PRS and global cortical volume, between global cortical volume and both negative and positive urgency, a significant positive association between fractional anisotropy and executive function, and a significant positive association between negative urgency and 1-year BMI SDS change (Table 3, Fig. 3). Together, these associations illustrate a potential pathway by which BMI-PRS could affect BMI SDS change via brain alterations that are related to higher impulsivity.

**Table 3:** Structural equation model results.

Outcome variable	Predictor	Estimate	Standard error	z-value	*p*-value
Cortical volume	BMI-PRS	−0.027	0.012	−2.242	0.025
Cortical thickness	BMI-PRS	−0.011	0.013	−0.818	0.414
Fractional anisotropy	BMI-PRS	0.003	0.011	0.229	0.819
Executive function	Cortical volume	−0.028	0.023	−1.251	0.211
	Cortical thickness	−0.027	0.019	−1.424	0.154
	Fractional anisotropy	0.100	0.020	5.041	0.000
Positive urgency	Cortical volume	−0.156	0.068	−2.282	0.022
	Cortical thickness	0.051	0.059	0.860	0.390
	Fractional anisotropy	0.030	0.060	0.503	0.615
Negative urgency	Cortical volume	−0.139	0.062	−2.249	0.025
	Cortical thickness	0.007	0.054	0.128	0.898
	Fractional anisotropy	−0.087	0.057	−1.537	0.124
BMI SDS increase	Executive function	−0.007	0.010	−0.735	0.462
	Positive urgency	0.002	0.004	0.472	0.637
	Negative urgency	0.010	0.004	2.440	0.015

*BMI-PRS* body mass index polygenic risk score. *BMI SDS* body mass index standard deviation score.

### Association between impulsivity and regional cortical volumes

To follow-up on significant associations between negative and positive urgency and global cortical volume, we investigated how regional cortical volume is related to impulsivity. This analysis showed that impulsivity, much like BMI-PRS, was associated with lower cortical volume in fronto-temporal brain areas (Table 4). This strengthens the evidence for the mechanisms by which BMI-PRS could influence weight gain via effects on fronto-temporal brain areas that could subsequently influence impulsivity and weight gain.

**Table 4:** Association between positive/negative urgency and cortical volume.

Parcel	Hemisphere	Regression estimate	*p*-value
Positive urgency			
Caudal middle frontal	Left	−0.021	<0.001
Inferior temporal		−0.009	0.006
Rostral anterior cingulate		−0.017	<0.001
Superior frontal		−0.015	<0.001
Middle temporal	Right	−0.009	0.020
Transverse temporal		−0.008	<0.001
Insula		−0.019	<0.001
Negative urgency			
Caudal middle frontal	Left	−0.018	<0.001
Cuneus		−0.014	<0.001
Rostral middle frontal		−0.010	<0.001
Superior parietal		−0.008	0.002
Superior temporal		−0.015	0.014
Caudal anterior cingulate	Right	−0.013	<0.001
Inferior temporal		−0.013	<0.001
Precentral		−0.008	<0.001
Rostral anterior cingulate		−0.017	<0.001

## Discussion

Obesity is heritable and highly polygenic, and most of the risk alleles identified act in the central nervous system [15–17, 19]. Environmental factors are thought to interact with a vulnerable brain to favour weight gain, a theory supported by evidence that genetic predisposition to obesity is exacerbated in an obesogenic environment [17, 70]. Studies in adults have linked elevated BMI to executive function, impulsivity, and neuroanatomical changes, all of which have been argued to impact feeding behaviour [5, 14, 25, 50, 54–56, 71]. However, the direction of causality is unclear as chronic adiposity also leads to widespread changes in grey and white matter, and consequent cognitive changes [72]. We aimed to reduce the role of this possible confound by studying the effect of genetic risk rather than BMI per se in young children.

The risk alleles for obesity were normally distributed in our population, and the number of risk alleles per individual was linearly proportional to BMI SDS. Both of these findings were also observed in the Locke et al. GWAS study of 330,000 adults [15]. The small magnitude of the association between BMI SDS and BMI-PRS might mean that risk score for adult obesity is not very strongly related to childhood BMI. Nevertheless, it still suggests that the genetic predisposition for obesity is already influencing BMI before age 10, even if to a small degree. We further found effects of BMI-PRS on grey matter morphometry, supporting the proposition that these genes exert their obesogenic effects via the brain.

High BMI-PRS was related to differences in grey matter volume and thickness relative to age-expected values. More specifically, BMI-PRS was associated with reduced grey matter volume and cortical thickness in bilateral fronto-temporal areas. Some of these regions are associated with executive function and have long been implicated in the control of food intake and impulsivity in adults [24, 73–75]. Our findings raise the possibility that cognitive control and impulsivity are also implicated in childhood body weight. Indeed, changes in brain morphometry were also associated with both a composite score for executive function and personality measures of impulsivity (positive and negative urgency), while impulsivity was related to an increase in BMI SDS. The fact that the heteromodal and unimodal levels of sensory processing hierarchies were most affected by BMI-PRS is consistent with impulsive individuals exhibiting a non-normative maturation of the heteromodal regions [64, 76]. It is also in line with our previous findings relating those heteromodal and unimodal regions to BMI in adults [77]. Overall, this suggests that impulsivity and fronto-temporal cortical morphometry are linked to obesity in young children, and that the effect may mediate some of the genetic risk for obesity. Importantly, grey matter change has been associated with obesity itself in adults, not obesity risk, although there it is thought to reflect damage secondary to metabolic consequences of adiposity [25]. While we cannot rule out such an effect in 9 to 10-year-olds studied here, a more likely explanation may be that polygenic risk for obesity is associated with brain development. On the other hand, a lack of association between BMI-PRS and FA in our study might mean that genetic risk for adult obesity is not directly expressed in white matter microstructure in children, but rather that commonly reported associations between obesity and FA in adults are due to a detrimental effect of chronic adiposity on white matter integrity.

The influence of impulsivity but lack of influence of executive function on body weight in this population warrants some discussion. The links between obesity and reduced executive function have been established repeatedly in adults and children, even in the ABCD sample [50, 56]. Indeed, uncontrolled eating, a heritable trait associated with BMI that encompasses many aspects of food intake regulation [14], is correlated to lower performance on executive function tests in adults and higher impulsivity [24, 78], although these effects are not always consistent [14, 79]. Most authors suggest that cognitive control plays a role in food intake via decision-making about portion size, food choice, and the like, however, it is unlikely that this type of cognitive control is relevant in children. As for impulsivity, the links with BMI are also well-established, even in children [54, 80–83]. Studies in infants and children have shown that genetic risk for obesity manifests predominantly as increased hunger, reduced satiety, food responsiveness, or uncontrolled eating [23, 84–87], some of which could be related to impulsivity [88]. This would be in line with our findings that show genetic influence on BMI SDS change via brain and impulsivity measures.

A major limitation of our study and, perhaps, all studies investigating genetic risk for obesity in the ABCD sample, is the fact the BMI and BMI-PRS were not equally distributed over different study sites. BMI and BMI-PRS were also correlated with genetic principal components that reflect population stratification and that also differed by study site. While it is likely that the site differences in BMI-PRS also reflect population stratification, the differences in BMI might be related to other phenomena, such as different obesogenic environments, which is not considered as a confounding factor in our analysis. Thus, removing variance associated with genetic principal components could also remove some of the true associations between BMI-PRS or BMI and neurobehavioural data. Therefore, our study might not reflect all true associations between the investigated obesity-related features. Further, our study is predominantly cross-sectional and so does not allow us to properly investigate causality in associations between BMI-PRS, brain structure, executive function, and impulsivity. Finally, we also did not directly investigate any aspect of eating behavior.

The current research could support genetic risk for obesity as causally related to developmental brain changes, leading to excess weight accumulation via higher impulsivity and eating-related traits that are heritable and already expressed in infancy. This research contributes to a better understanding of neural mechanisms of obesity in adolescence and could inspire future strategies for obesity prevention. Because BMI-PRS can be calculated early in life, individuals at high risk for obesity could be identified and targeted by interventions that are, e.g., aimed at decreasing impulsivity, which could lead to beneficial health outcomes in the future.
